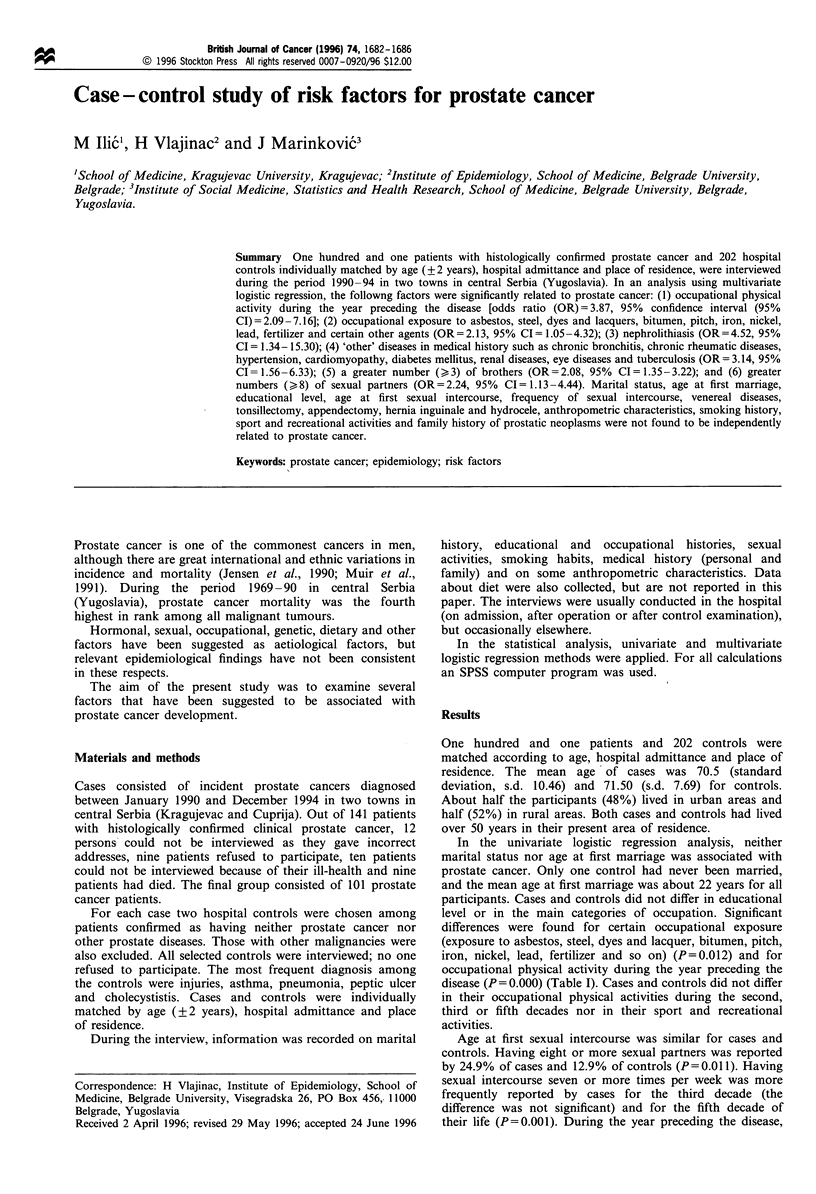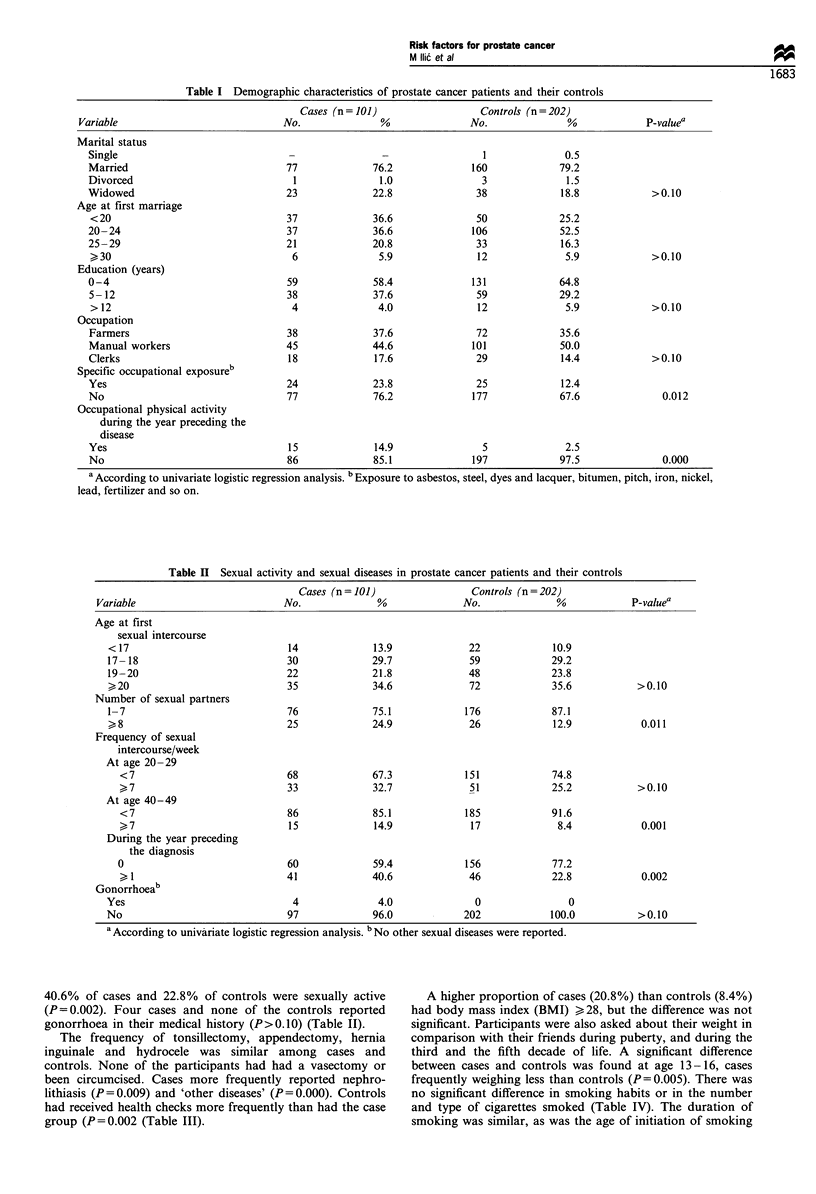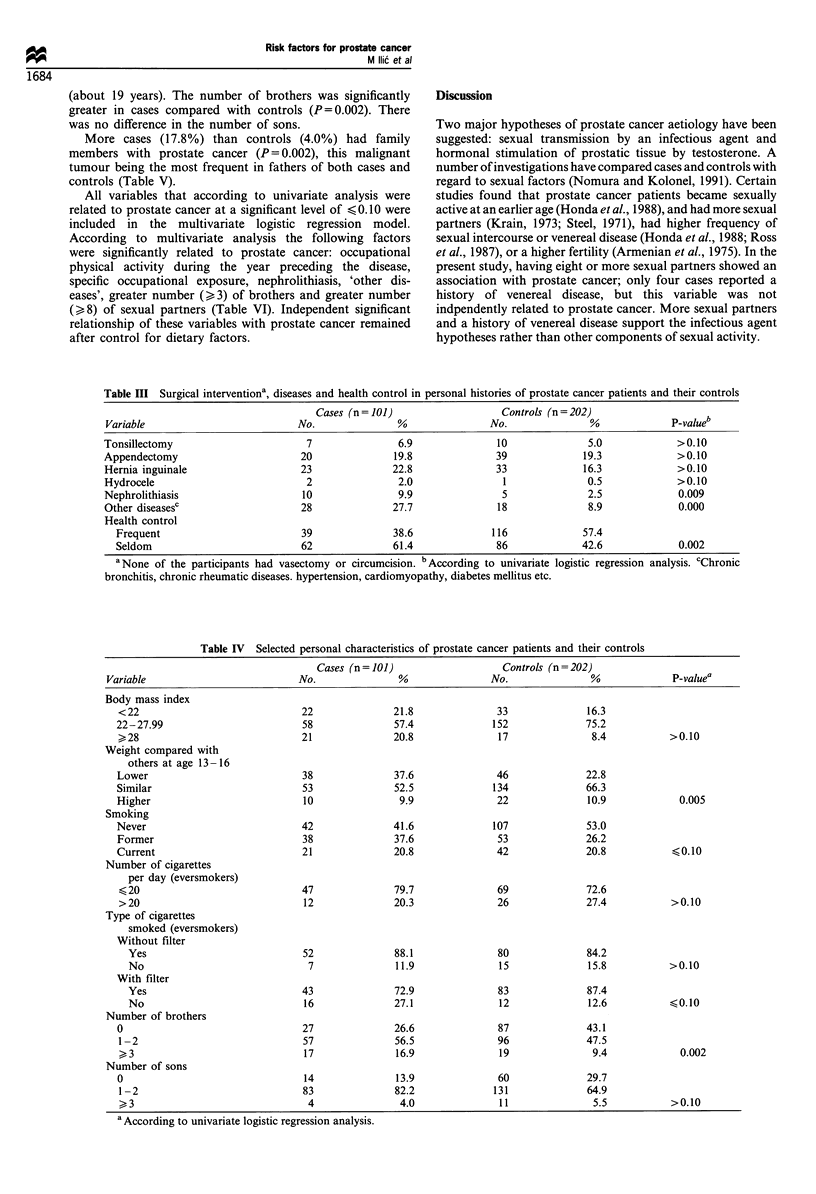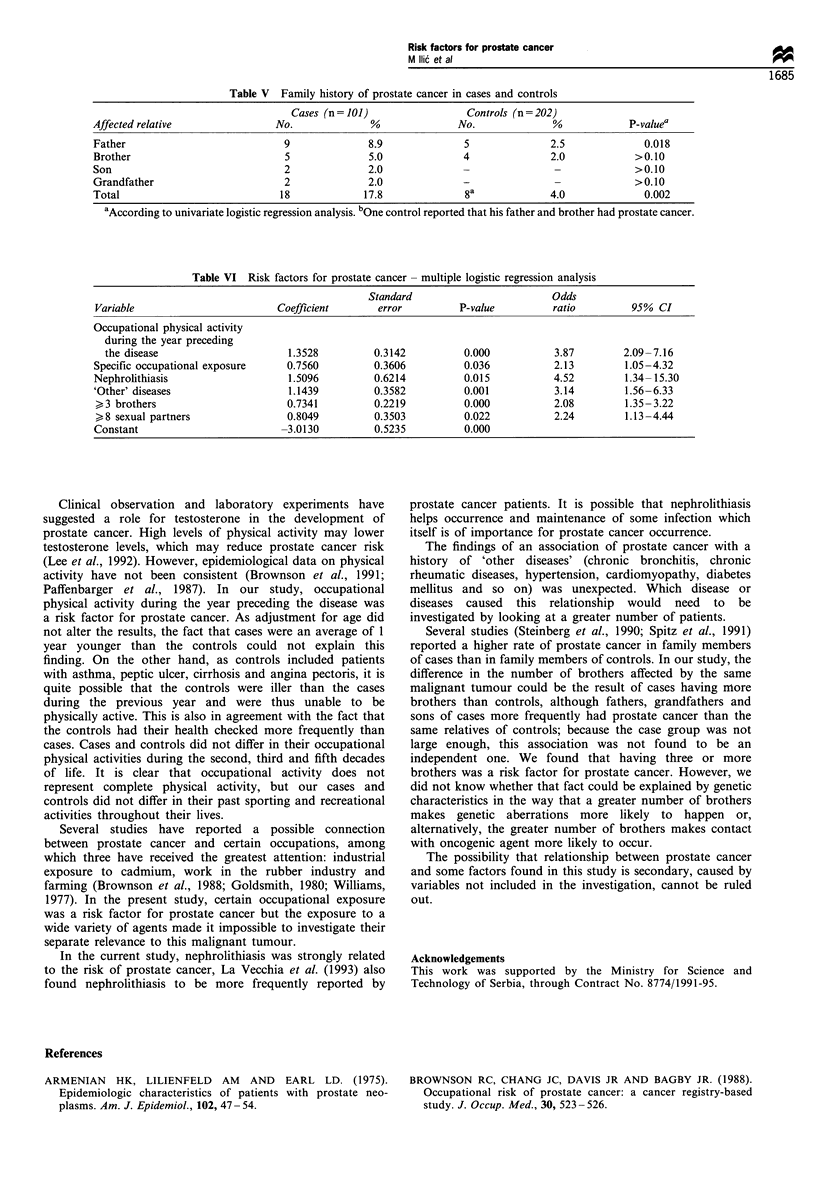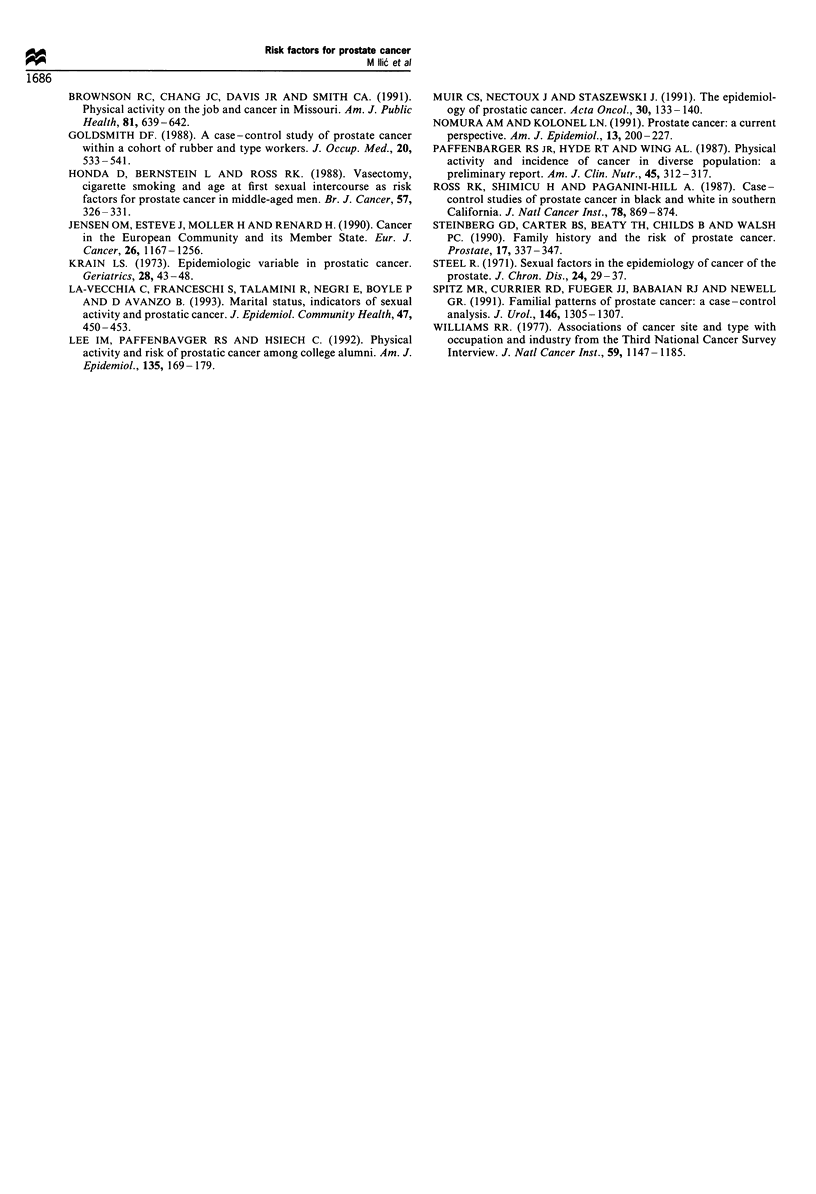# Case-control study of risk factors for prostate cancer.

**DOI:** 10.1038/bjc.1996.610

**Published:** 1996-11

**Authors:** M. Ilić, H. Vlajinac, J. Marinković

**Affiliations:** School of Medicine, Kragujevac University, Yugoslavia.

## Abstract

One hundred and one patients with histologically confirmed prostate cancer and 202 hospital controls individually matched by age (+/- 2 years), hospital admittance and place of residence, were interviewed during the period 1990-94 in two towns in central Serbia (Yugoslavia). In an analysis using multivariate logistic regression, the followng factors were significantly related to prostate cancer: (1) occupational physical activity during the year preceding the disease [odds ratio (OR)=3.87, 95% confidence interval (95% CI)=2.09-7.16]; (2) occupational exposure to asbestos, steel, dyes and lacquers, bitumen, pitch, iron, nickel, lead, fertilizer and certain other agents (OR=2.13, 95% CI=1.05-4.32); (3) nephrolithiasis (OR=4.52, 95% CI=1.34-15.30); (4) 'other' diseases in medical history such as chronic bronchitis, chronic rheumatic diseases, hypertension, cardiomyopathy, diabetes mellitus, renal diseases, eye diseases and tuberculosis (OR=3.14, 95% CI=1.56-6.33); (5) a greater number (> or = 3) of brothers (OR=2.08, 95% CI=1.35-3.22); and (6) greater numbers (> or = 8) of sexual partners (OR=2.24, 95% CI=1.13-4.44). Marital status, age at first marriage, educational level, age at first sexual intercourse, frequency of sexual intercourse, venereal diseases, tonsillectomy, appendectomy, hernia inguinale and hydrocele, anthropometric characteristics, smoking history, sport and recreational activities and family history of prostatic neoplasms were not found to be independently related to prostate cancer.


British Journal of Cancer (1996) 74, 1682-1686
© 1996 Stockton Press All rights reserved 0007-0920/96 $12.00

### Case-control study of risk factors for prostate cancer

M Ilić[1], H Vlajinac[2] and J Marinković[3]

[1]School of Medicine, Kragujevac University, Kragujevac; [2]Institute of Epidemiology, School of Medicine, Belgrade University, Belgrade; [3]Institute of Social Medicine, Statistics and Health Research, School of Medicine, Belgrade University, Belgrade, Yugoslavia.

**Summary** One hundred and one patients with histologically confirmed prostate cancer and 202 hospital controls individually matched by age (±2 years), hospital admittance and place of residence, were interviewed during the period 1990-94 in two towns in central Serbia (Yugoslavia). In an analysis using multivariate logistic regression, the followng factors were significantly related to prostate cancer: (1) occupational physical activity during the year preceding the disease [odds ratio (OR)=3.87, 95% confidence interval (95% CI)=2.09-7.16]; (2) occupational exposure to asbestos, steel, dyes and lacquers, bitumen, pitch, iron, nickel, lead, fertilizer and certain other agents (OR=2.13, 95% CI=1.05-4.32); (3) nephrolithiasis (OR=4.52, 95% CI=1.34-15.30); (4) 'other' diseases in medical history such as chronic bronchitis, chronic rheumatic diseases, hypertension, cardiomyopathy, diabetes mellitus, renal diseases, eye diseases and tuberculosis (OR=3.14, 95% CI=1.56-6.33); (5) a greater number (⩾3) of brothers (OR=2.08, 95% CI=1.35-3.22); and (6) greater numbers (⩾8) of sexual partners (OR=2.24, 95% CI=1.13-4.44). Marital status, age at first marriage, educational level, age at first sexual intercourse, frequency of sexual intercourse, venereal diseases, tonsillectomy, appendectomy, hernia inguinale and hydrocele, anthropometric characteristics, smoking history, sport and recreational activities and family history of prostatic neoplasms were not found to be independently related to prostate cancer.

**Keywords:** prostate cancer; epidemiology; risk factors

Prostate cancer is one of the commonest cancers in men, although there are great international and ethnic variations in incidence and mortality (Jensen *et al.*, 1990; Muir *et al.*, 1991). During the period 1969-90 in central Serbia (Yugoslavia), prostate cancer mortality was the fourth highest in rank among all malignant tumours.

Hormonal, sexual, occupational, genetic, dietary and other factors have been suggested as aetiological factors, but relevant epidemiological findings have not been consistent in these respects.

The aim of the present study was to examine several factors that have been suggested to be associated with prostate cancer development.

### Materials and methods

Cases consisted of incident prostate cancers diagnosed between January 1990 and December 1994 in two towns in central Serbia (Kragujevac and Cuprija). Out of 141 patients with histologically confirmed clinical prostate cancer, 12 persons could not be interviewed as they gave incorrect addresses, nine patients refused to participate, ten patients could not be interviewed because of their ill-health and nine patients had died. The final group consisted of 101 prostate cancer patients.

For each case two hospital controls were chosen among patients confirmed as having neither prostate cancer nor other prostate diseases. Those with other malignancies were also excluded. All selected controls were interviewed; no one refused to participate. The most frequent diagnosis among the controls were injuries, asthma, pneumonia, peptic ulcer and cholecystistis. Cases and controls were individually matched by age (±2 years), hospital admittance and place of residence.

During the interview, information was recorded on marital history, educational and occupational histories, sexual activities, smoking habits, medical history (personal and family) and on some anthropometric characteristics. Data about diet were also collected, but are not reported in this paper. The interviews were usually conducted in the hospital (on admission, after operation or after control examination), but occasionally elsewhere.

In the statistical analysis, univariate and multivariate logistic regression methods were applied. For all calculations an SPSS computer program was used.

Correspondence: H Vlajinac, Institute of Epidemiology, School of Medicine, Belgrade University, Visegradska 26, PO Box 456, 11000 Belgrade, Yugoslavia
Received 2 April 1996; revised 29 May 1996; accepted 24 June 1996

### Results

One hundred and one patients and 202 controls were matched according to age, hospital admittance and place of residence. The mean age of cases was 70.5 (standard deviation, s.d. 10.46) and 71.50 (s.d. 7.69) for controls. About half the participants (48%) lived in urban areas and half (52%) in rural areas. Both cases and controls had lived over 50 years in their present area of residence.

In the univariate logistic regression analysis, neither marital status nor age at first marriage was associated with prostate cancer. Only one control had never been married, and the mean age at first marriage was about 22 years for all participants. Cases and controls did not differ in educational level or in the main categories of occupation. Significant differences were found for certain occupational exposure (exposure to asbestos, steel, dyes and lacquer, bitumen, pitch, iron, nickel, lead, fertilizer and so on) ($P=0.012$) and for occupational physical activity during the year preceding the disease ($P=0.000$) (Table I). Cases and controls did not differ in their occupational physical activities during the second, third or fifth decades nor in their sport and recreational activities.

Age at first sexual intercourse was similar for cases and controls. Having eight or more sexual partners was reported by 24.9% of cases and 12.9% of controls ($P=0.011$). Having sexual intercourse seven or more times per week was more frequently reported by cases for the third decade (the difference was not significant) and for the fifth decade of their life ($P=0.001$). During the year preceding the disease,

Table I Demographic characteristics of prostate cancer patients and their controls

| Variable | Cases (n=101) No. | % | Controls (n=202) No. | % | P-value[a] |
|---|---|---|---|---|---|
| Marital status | | | | | |
| Single | – | – | 1 | 0.5 | |
| Married | 77 | 76.2 | 160 | 79.2 | |
| Divorced | 1 | 1.0 | 3 | 1.5 | |
| Widowed | 23 | 22.8 | 38 | 18.8 | >0.10 |
| Age at first marriage | | | | | |
| <20 | 37 | 36.6 | 50 | 25.2 | |
| 20–24 | 37 | 36.6 | 106 | 52.5 | |
| 25–29 | 21 | 20.8 | 33 | 16.3 | |
| ⩾30 | 6 | 5.9 | 12 | 5.9 | >0.10 |
| Education (years) | | | | | |
| 0–4 | 59 | 58.4 | 131 | 64.8 | |
| 5–12 | 38 | 37.6 | 59 | 29.2 | |
| >12 | 4 | 4.0 | 12 | 5.9 | >0.10 |
| Occupation | | | | | |
| Farmers | 38 | 37.6 | 72 | 35.6 | |
| Manual workers | 45 | 44.6 | 101 | 50.0 | |
| Clerks | 18 | 17.6 | 29 | 14.4 | >0.10 |
| Specific occupational exposure[b] | | | | | |
| Yes | 24 | 23.8 | 25 | 12.4 | |
| No | 77 | 76.2 | 177 | 67.6 | 0.012 |
| Occupational physical activity during the year preceding the disease | | | | | |
| Yes | 15 | 14.9 | 5 | 2.5 | |
| No | 86 | 85.1 | 197 | 97.5 | 0.000 |

[a] According to univariate logistic regression analysis. [b] Exposure to asbestos, steel, dyes and lacquer, bitumen, pitch, iron, nickel, lead, fertilizer and so on.

Table II Sexual activity and sexual diseases in prostate cancer patients and their controls

| Variable | Cases (n=101) No. | % | Controls (n=202) No. | % | P-value[a] |
|---|---|---|---|---|---|
| Age at first sexual intercourse | | | | | |
| <17 | 14 | 13.9 | 22 | 10.9 | |
| 17–18 | 30 | 29.7 | 59 | 29.2 | |
| 19–20 | 22 | 21.8 | 48 | 23.8 | |
| ⩾20 | 35 | 34.6 | 72 | 35.6 | >0.10 |
| Number of sexual partners | | | | | |
| 1–7 | 76 | 75.1 | 176 | 87.1 | |
| ⩾8 | 25 | 24.9 | 26 | 12.9 | 0.011 |
| Frequency of sexual intercourse/week | | | | | |
| At age 20–29 | | | | | |
| <7 | 68 | 67.3 | 151 | 74.8 | |
| ⩾7 | 33 | 32.7 | 51 | 25.2 | >0.10 |
| At age 40–49 | | | | | |
| <7 | 86 | 85.1 | 185 | 91.6 | |
| ⩾7 | 15 | 14.9 | 17 | 8.4 | 0.001 |
| During the year preceding the diagnosis | | | | | |
| 0 | 60 | 59.4 | 156 | 77.2 | |
| ⩾1 | 41 | 40.6 | 46 | 22.8 | 0.002 |
| Gonorrhoea[b] | | | | | |
| Yes | 4 | 4.0 | 0 | 0 | |
| No | 97 | 96.0 | 202 | 100.0 | >0.10 |

[a] According to univariate logistic regression analysis. [b] No other sexual diseases were reported.

40.6% of cases and 22.8% of controls were sexually active ($P$=0.002). Four cases and none of the controls reported gonorrhoea in their medical history ($P$>0.10) (Table II).

The frequency of tonsillectomy, appendectomy, hernia inguinale and hydrocele was similar among cases and controls. None of the participants had had a vasectomy or been circumcised. Cases more frequently reported nephrolithiasis ($P$=0.009) and 'other diseases' ($P$=0.000). Controls had received health checks more frequently than had the case group ($P$=0.002 (Table III).

A higher proportion of cases (20.8%) than controls (8.4%) had body mass index (BMI) ⩾28, but the difference was not significant. Participants were also asked about their weight in comparison with their friends during puberty, and during the third and the fifth decade of life. A significant difference between cases and controls was found at age 13–16, cases frequently weighing less than controls ($P$=0.005). There was no significant difference in smoking habits or in the number and type of cigarettes smoked (Table IV). The duration of smoking was similar, as was the age of initiation of smoking

(about 19 years). The number of brothers was significantly greater in cases compared with controls ($P=0.002$). There was no difference in the number of sons.

More cases (17.8%) than controls (4.0%) had family members with prostate cancer ($P=0.002$), this malignant tumour being the most frequent in fathers of both cases and controls (Table V).

All variables that according to univariate analysis were related to prostate cancer at a significant level of ⩽0.10 were included in the multivariate logistic regression model. According to multivariate analysis the following factors were significantly related to prostate cancer: occupational physical activity during the year preceding the disease, specific occupational exposure, nephrolithiasis, 'other diseases', greater number (⩾3) of brothers and greater number (⩾8) of sexual partners (Table VI). Independent significant relationship of these variables with prostate cancer remained after control for dietary factors.

### Discussion

Two major hypotheses of prostate cancer aetiology have been suggested: sexual transmission by an infectious agent and hormonal stimulation of prostatic tissue by testosterone. A number of investigations have compared cases and controls with regard to sexual factors (Nomura and Kolonel, 1991). Certain studies found that prostate cancer patients became sexually active at an earlier age (Honda *et al.*, 1988), and had more sexual partners (Krain, 1973; Steel, 1971), had higher frequency of sexual intercourse or venereal disease (Honda *et al.*, 1988; Ross *et al.*, 1987), or a higher fertility (Armenian *et al.*, 1975). In the present study, having eight or more sexual partners showed an association with prostate cancer; only four cases reported a history of venereal disease, but this variable was not indpendently related to prostate cancer. More sexual partners and a history of venereal disease support the infectious agent hypotheses rather than other components of sexual activity.

**Table III** Surgical intervention[a], diseases and health control in personal histories of prostate cancer patients and their controls

| Variable | Cases (n=101) No. | % | Controls (n=202) No. | % | P-value[b] |
|---|---|---|---|---|---|
| Tonsillectomy | 7 | 6.9 | 10 | 5.0 | >0.10 |
| Appendectomy | 20 | 19.8 | 39 | 19.3 | >0.10 |
| Hernia inguinale | 23 | 22.8 | 33 | 16.3 | >0.10 |
| Hydrocele | 2 | 2.0 | 1 | 0.5 | >0.10 |
| Nephrolithiasis | 10 | 9.9 | 5 | 2.5 | 0.009 |
| Other diseases[c] | 28 | 27.7 | 18 | 8.9 | 0.000 |
| Health control | | | | | |
| Frequent | 39 | 38.6 | 116 | 57.4 | |
| Seldom | 62 | 61.4 | 86 | 42.6 | 0.002 |

[a]None of the participants had vasectomy or circumcision. [b]According to univariate logistic regression analysis. [c]Chronic bronchitis, chronic rheumatic diseases. hypertension, cardiomyopathy, diabetes mellitus etc.

**Table IV** Selected personal characteristics of prostate cancer patients and their controls

| Variable | Cases (n=101) No. | % | Controls (n=202) No. | % | P-value[a] |
|---|---|---|---|---|---|
| Body mass index | | | | | |
| <22 | 22 | 21.8 | 33 | 16.3 | |
| 22–27.99 | 58 | 57.4 | 152 | 75.2 | |
| ⩾28 | 21 | 20.8 | 17 | 8.4 | >0.10 |
| Weight compared with others at age 13–16 | | | | | |
| Lower | 38 | 37.6 | 46 | 22.8 | |
| Similar | 53 | 52.5 | 134 | 66.3 | |
| Higher | 10 | 9.9 | 22 | 10.9 | 0.005 |
| Smoking | | | | | |
| Never | 42 | 41.6 | 107 | 53.0 | |
| Former | 38 | 37.6 | 53 | 26.2 | |
| Current | 21 | 20.8 | 42 | 20.8 | ⩽0.10 |
| Number of cigarettes per day (eversmokers) | | | | | |
| ⩽20 | 47 | 79.7 | 69 | 72.6 | |
| >20 | 12 | 20.3 | 26 | 27.4 | >0.10 |
| Type of cigarettes smoked (eversmokers) | | | | | |
| Without filter | | | | | |
| Yes | 52 | 88.1 | 80 | 84.2 | |
| No | 7 | 11.9 | 15 | 15.8 | >0.10 |
| With filter | | | | | |
| Yes | 43 | 72.9 | 83 | 87.4 | |
| No | 16 | 27.1 | 12 | 12.6 | ⩽0.10 |
| Number of brothers | | | | | |
| 0 | 27 | 26.6 | 87 | 43.1 | |
| 1–2 | 57 | 56.5 | 96 | 47.5 | |
| ⩾3 | 17 | 16.9 | 19 | 9.4 | 0.002 |
| Number of sons | | | | | |
| 0 | 14 | 13.9 | 60 | 29.7 | |
| 1–2 | 83 | 82.2 | 131 | 64.9 | |
| ⩾3 | 4 | 4.0 | 11 | 5.5 | >0.10 |

[a] According to univariate logistic regression analysis.

Table V Family history of prostate cancer in cases and controls

| Affected relative | Cases (n=101) No. | % | Controls (n=202) No. | % | P-value[a] |
|---|---|---|---|---|---|
| Father | 9 | 8.9 | 5 | 2.5 | 0.018 |
| Brother | 5 | 5.0 | 4 | 2.0 | >0.10 |
| Son | 2 | 2.0 | – | – | >0.10 |
| Grandfather | 2 | 2.0 | – | – | >0.10 |
| Total | 18 | 17.8 | 8[a] | 4.0 | 0.002 |

[a]According to univariate logistic regression analysis. [b]One control reported that his father and brother had prostate cancer.

Table VI Risk factors for prostate cancer – multiple logistic regression analysis

| Variable | Coefficient | Standard error | P-value | Odds ratio | 95% CI |
|---|---|---|---|---|---|
| Occupational physical activity during the year preceding the disease | 1.3528 | 0.3142 | 0.000 | 3.87 | 2.09–7.16 |
| Specific occupational exposure | 0.7560 | 0.3606 | 0.036 | 2.13 | 1.05–4.32 |
| Nephrolithiasis | 1.5096 | 0.6214 | 0.015 | 4.52 | 1.34–15.30 |
| 'Other' diseases | 1.1439 | 0.3582 | 0.001 | 3.14 | 1.56–6.33 |
| ⩾3 brothers | 0.7341 | 0.2219 | 0.000 | 2.08 | 1.35–3.22 |
| ⩾8 sexual partners | 0.8049 | 0.3503 | 0.022 | 2.24 | 1.13–4.44 |
| Constant | -3.0130 | 0.5235 | 0.000 | | |

Clinical observation and laboratory experiments have suggested a role for testosterone in the development of prostate cancer. High levels of physical activity may lower testosterone levels, which may reduce prostate cancer risk (Lee *et al.*, 1992). However, epidemiological data on physical activity have not been consistent (Brownson *et al.*, 1991; Paffenbarger *et al.*, 1987). In our study, occupational physical activity during the year preceding the disease was a risk factor for prostate cancer. As adjustment for age did not alter the results, the fact that cases were an average of 1 year younger than the controls could not explain this finding. On the other hand, as controls included patients with asthma, peptic ulcer, cirrhosis and angina pectoris, it is quite possible that the controls were iller than the cases during the previous year and were thus unable to be physically active. This is also in agreement with the fact that the controls had their health checked more frequently than cases. Cases and controls did not differ in their occupational physical activities during the second, third and fifth decades of life. It is clear that occupational activity does not represent complete physical activity, but our cases and controls did not differ in their past sporting and recreational activities throughout their lives.

Several studies have reported a possible connection between prostate cancer and certain occupations, among which three have received the greatest attention: industrial exposure to cadmium, work in the rubber industry and farming (Brownson *et al.*, 1988; Goldsmith, 1980; Williams, 1977). In the present study, certain occupational exposure was a risk factor for prostate cancer but the exposure to a wide variety of agents made it impossible to investigate their separate relevance to this malignant tumour.

In the current study, nephrolithiasis was strongly related to the risk of prostate cancer, La Vecchia *et al.* (1993) also found nephrolithiasis to be more frequently reported by prostate cancer patients. It is possible that nephrolithiasis helps occurrence and maintenance of some infection which itself is of importance for prostate cancer occurrence.

The findings of an association of prostate cancer with a history of 'other diseases' (chronic bronchitis, chronic rheumatic diseases, hypertension, cardiomyopathy, diabetes mellitus and so on) was unexpected. Which disease or diseases caused this relationship would need to be investigated by looking at a greater number of patients.

Several studies (Steinberg *et al.*, 1990; Spitz *et al.*, 1991) reported a higher rate of prostate cancer in family members of cases than in family members of controls. In our study, the difference in the number of brothers affected by the same malignant tumour could be the result of cases having more brothers than controls, although fathers, grandfathers and sons of cases more frequently had prostate cancer than the same relatives of controls; because the case group was not large enough, this association was not found to be an independent one. We found that having three or more brothers was a risk factor for prostate cancer. However, we did not know whether that fact could be explained by genetic characteristics in the way that a greater number of brothers makes genetic aberrations more likely to happen or, alternatively, the greater number of brothers makes contact with oncogenic agent more likely to occur.

The possibility that relationship between prostate cancer and some factors found in this study is secondary, caused by variables not included in the investigation, cannot be ruled out.

**Acknowledgements**
This work was supported by the Ministry for Science and Technology of Serbia, through Contract No. 8774/1991-95.

### References

ARMENIAN HK, LILIENFELD AM AND EARL LD. (1975). Epidemiologic characteristics of patients with prostate neoplasms. *Am. J. Epidemiol.*, **102**, 47–54.

BROWNSON RC, CHANG JC, DAVIS JR AND BAGBY JR. (1988). Occupational risk of prostate cancer: a cancer registry-based study. *J. Occup. Med.*, **30**, 523–526.

BROWNSON RC, CHANG JC, DAVIS JR AND SMITH CA. (1991). Physical activity on the job and cancer in Missouri. *Am. J. Public Health*, **81,** 639–642.

GOLDSMITH DF. (1988). A case–control study of prostate cancer within a cohort of rubber and type workers. *J. Occup. Med.*, **20,** 533–541.

HONDA D, BERNSTEIN L AND ROSS RK. (1988). Vasectomy, cigarette smoking and age at first sexual intercourse as risk factors for prostate cancer in middle-aged men. *Br. J. Cancer*, **57,** 326–331.

JENSEN OM, ESTEVE J, MOLLER H AND RENARD H. (1990). Cancer in the European Community and its Member State. *Eur. J. Cancer*, **26,** 1167–1256.

KRAIN LS. (1973). Epidemiologic variable in prostatic cancer. *Geriatrics*, **28,** 43–48.

LA-VECCHIA C, FRANCESCHI S, TALAMINI R, NEGRI E, BOYLE P AND D AVANZO B. (1993). Marital status, indicators of sexual activity and prostatic cancer. *J. Epidemiol. Community Health*, **47,** 450–453.

LEE IM, PAFFENBAVGER RS AND HSIECH C. (1992). Physical activity and risk of prostatic cancer among college alumni. *Am. J. Epidemiol.*, **135,** 169–179.

MUIR CS, NECTOUX J AND STASZEWSKI J. (1991). The epidemiology of prostatic cancer. *Acta Oncol.*, **30,** 133–140.

NOMURA AM AND KOLONEL LN. (1991). Prostate cancer: a current perspective. *Am. J. Epidemiol.*, **13,** 200–227.

PAFFENBARGER RS JR, HYDE RT AND WING AL. (1987). Physical activity and incidence of cancer in diverse population: a preliminary report. *Am. J. Clin. Nutr.*, **45,** 312–317.

ROSS RK, SHIMICU H AND PAGANINI-HILL A. (1987). Case–control studies of prostate cancer in black and white in southern California. *J. Natl Cancer Inst.*, **78,** 869–874.

STEINBERG GD, CARTER BS, BEATY TH, CHILDS B AND WALSH PC. (1990). Family history and the risk of prostate cancer. *Prostate*, **17,** 337–347.

STEEL R. (1971). Sexual factors in the epidemiology of cancer of the prostate. *J. Chron. Dis.*, **24,** 29–37.

SPITZ MR, CURRIER RD, FUEGER JJ, BABAIAN RJ AND NEWELL GR. (1991). Familial patterns of prostate cancer: a case–control analysis. *J. Urol.*, **146,** 1305–1307.

WILLIAMS RR. (1977). Associations of cancer site and type with occupation and industry from the Third National Cancer Survey Interview. *J. Natl Cancer Inst.*, **59,** 1147–1185.